# Rehabilitation Improves Mitochondrial Energetics in Progressive Multiple Sclerosis: The Significant Role of Robot-Assisted Gait Training and of the Personalized Intensity

**DOI:** 10.3390/diagnostics10100834

**Published:** 2020-10-17

**Authors:** Fabio Manfredini, Sofia Straudi, Nicola Lamberti, Simone Patergnani, Veronica Tisato, Paola Secchiero, Francesco Bernardi, Nicole Ziliotto, Giovanna Marchetti, Nino Basaglia, Massimo Bonora, Paolo Pinton

**Affiliations:** 1 Department of Neuroscience and rehabilitation, University of Ferrara, 44121 Ferrara, Italy; fabio.manfredini@unife.it (F.M.); giovanna.marchetti@unife.it (G.M.); 2 Department of Neuroscience/Rehabilitation, Unit of Rehabilitation Medicine, University Hospital of Ferrara, 44124 Ferrara, Italy; sofia.straudi@unife.it (S.S.); nino.basaglia@unife.it (N.B.); 3 Department of Medical Sciences, Laboratory for Technologies of Advanced Therapies (LTTA), University of Ferrara, 44121 Ferrara, Italy; simone.patergnani@unife.it (S.P.); massimo.bonora1@unife.it (M.B.); paolo.pinton@unife.it (P.P.); 4 Department of Morphology, Surgery and Experimental Medicine and LTTA Centre, University of Ferrara, 44121 Ferrara, Italy; veronica.tisato@unife.it (V.T.); paola.secchiero@unife.it (P.S.); 5 Department of Life Sciences and Biotechnology, University of Ferrara, 44121 Ferrara, Italy; francesco.bernardi@unife.it; 6 School of Medicine and Surgery, University of Milano-Bicocca, 20900 Monza, Italy; nicole.ziliotto@unimib.it

**Keywords:** multiple sclerosis, exercise training, lactic acid, pyruvic acid

## Abstract

Abnormal levels of pyruvate and lactate were reported in multiple sclerosis (MS). We studied the response of markers of mitochondrial function to rehabilitation in relation to type, intensity and endurance performance in severely disabled MS patients. Forty-six progressive MS patients were randomized to receive 12 walking sessions of robot-assisted gait training (RAGT, *n* = 23) or conventional overground therapy (CT, *n* = 23). Ten healthy subjects were also studied. Blood samples were collected to determine lactate, pyruvate, and glutathione levels and lactate/pyruvate ratio pre–post rehabilitation. In vivo muscle metabolism and endurance walking capacity were assessed by resting muscle oxygen consumption (rmVO_2_) using near-infrared spectroscopy and by six-minute walking distance (6MWD), respectively. The levels of mitochondrial biomarkers and rmVO_2_, altered at baseline with respect to healthy subjects, improved after rehabilitation in the whole population. In the two groups, an enhanced response was observed after RAGT compared to CT for lactate (*p* = 0.012), glutathione (<0.001), lactate/pyruvate ratio (*p* = 0.08) and rmVO_2_ (*p* = 0.07). Metabolic biomarkers and 6MWD improvements were exclusively correlated with a training speed markedly below individual gait speed. In severely disabled MS patients, rehabilitation rebalanced altered serum metabolic and muscle parameters, with RAGT being more effective than CT. A determinable slow training speed was associated with better metabolic and functional recovery. Trial Registration: ClinicalTrials.gov NCT02421731.

## 1. Introduction

Altered metabolic dynamics have frequently been observed in pathologic conditions and in several neurodegenerative disorders [1,2]. In the last decade, several studies have associated biochemical changes (ATP production, oxygen levels and glucose availability) with multiple sclerosis (MS), a chronic inflammatory disease affecting the central nervous system [3,4].

Aerobic metabolism and the degradation of substrates, in particular glucose, are crucial for energy production considering that the brain utilizes approximately 20% of both the body’s daily glucose consumption [5] and that muscle can increase resting glucose uptake as much as 30 to 50 times during high intensity exercise [6].

Glucose catabolism through glycolysis produces pyruvate, which, under aerobic conditions, is oxidized in the mitochondria to produce 36 ATPs through the tricarboxylic acid cycle and oxidative phosphorylation [7]. Pyruvate is reduced to lactate in the cytoplasm and then secreted into the extracellular space [7] in the presence of low oxygen availability or, in general, when pyruvate formation exceeds pyruvate oxidation, including in a state of low mitochondrial oxidative capacity [8]. Lactate production also occurs in so-called inflammatory hypoxia [9].

During the respiration process, a proportion of oxygen is converted to reactive oxygen species [10,11] (ROS), which, in general, are offset by protective antioxidants such as cellular enzymes and vitamins. An excess of ROS production may alter redox homeostasis and induce oxidative stress, which modulates cell cycle progression, cell survival and apoptosis [11,12,13]. Mitochondrial dysfunction, altered energetic metabolism and excessive ROS production represent potential pathogenic factors contributing to several diseases, including neurological disorders [1,2,14].

Abnormal values of pyruvate [3], increased anaerobic metabolism even in normoxic conditions [9,15], and high levels of lactate and mitochondrial impairments have been reported in MS lesions and in the cerebrospinal fluid and serum of MS patients [3,16,17,18,19]. Interestingly, all these factors are frequently associated with inflammatory responses and worsening or progression of disease [17,18,19,20].

To demonstrate the presence of mitochondrial dysfunction during the progression of MS, we recently found excessive amounts of mitophagy markers in MS patients in the active phase of the disease. Notably, mitophagy is a mechanism responsible for helping preserve a functional mitochondrial population. If expressed at higher levels, the results are detrimental for the cell, provoking energetic deficits [18,19].

Serum lactate levels, which have been found to be abnormally high in patients with MS and correlated with the severity of disease [15], were considered a potentially useful marker for monitoring “virtual hypoxia”, metabolic dysfunction and disease progression [3,15]. However, it is known that abnormal lactate levels may be connected to disease-related or disuse-induced changes in muscle [5]. Mitochondrial dysfunction and lower oxidative capacity [21,22,23] are also critical factors in the performance of skeletal muscles, where aerobic metabolism is sustained by networks of mitochondria [21]. Compared with healthy controls, patients with MS have a higher resting muscle oxygen consumption [24], higher respiratory quotient [22], greater energy expenditure after light exercise [25], earlier lactate production and lower aerobic power [15,25,26], with a negative impact on exercise tolerance and mobility [25,27]. It is known that exercise training improves mitochondrial function in skeletal muscle, causing lower levels of fatigue and higher endurance performance [28], possibly by stimulating the same signaling pathways in the brain [21,28,29]. However, a different dose (frequency, intensity, duration and type [30]) of exercise in the same subject or the same dose in different subjects may have different effects in terms of energetic metabolism and plastic adaptations (muscle fiber type response, production of neurotrophic factors, etc.) [31].

We hypothesized that rehabilitative exercise might significantly improve the energetic status of persons with MS and that different effects might be associated with the type and intensity of training. Recently, we completed a large trial aimed at comparing the effects of robot-assisted gait training (RAGT) and intensive overground walking (CT) on gait speed [32] in MS patients with higher degrees of disability [33]. One of the secondary aims of the RAGTIME study was to study clinical and circulating markers of plasticity [33] following rehabilitation.

The present study aimed (i) to explore the energetic metabolism of patients with severe MS using selected markers of mitochondrial function (lactate, pyruvate, the lactate/pyruvate ratio and glutathione, GSH) at baseline and in response to rehabilitation and (ii) to assess metabolic and endurance performance changes according to the type and intensity of exercise.

## 2. Materials and Methods

### 2.1. Subjects

The patients included in this study were a subsample (*n* = 46) of those participating in the RAGTIME trial, a randomized controlled study that compared two different walking rehabilitation protocols in progressive MS patients with severe disability (NCT02421731) [32]. The participants adhered to the following inclusion criteria: age between 18 and 65 years and progressive MS with severe gait impairments, defined by an expanded disability status scale (EDSS) score ranging from 6 to 7. Otherwise, subjects were excluded if one of the following conditions was present: inability to perform the timed 25-foot walk test; MS worsening in the 3 months before the intervention; impaired cognitive function; severe muscle spasticity; additional clinical conditions related to MS that may have interfered with the safe completion of the protocol; and changes in drug therapy during the study or receipt of other rehabilitation treatments or botulinum toxin injection [33]. In addition to the original criteria [33], patients who had missing values for any biomarkers included in this study at any collection time point were also excluded to avoid missing data. Finally, a population of healthy subjects (*n* = 10) without any known disease who were free from medications were included in the study for appropriate comparisons. The local ethics committee of Ferrara University Hospital approved the study (number 101-2012), approved on 27 September 2012. The research was carried out according to The Code of Ethics of the World Medical Association (Declaration of Helsinki), and written informed consent was obtained from all participants.

### 2.2. Outcome Measures

Outcome measures considered in this study were collected at baseline (T0), after 6 rehabilitation sessions (T1) and at the end of rehabilitation (T2). A single-blind analysis was employed, with outcome assessors and laboratory technicians blinded to group allocation.

### 2.3. Blood Collection and Determination of Mitochondrial Biomarkers

Blood collection: To standardize the biomarker collection and to limit any possible bias upon arrival of the patients at the rehabilitation unit, a team member picked the patients up using a wheelchair and transported them to the blood collection site. After 10 min of seated rest, using the standard tourniquet procedure, the same skilled operator collected blood from the antecubital vein into three 6 mL VACUETTE^®^ polypropylene tubes containing ethylenediaminetetraacetic acid (EDTA). Blood samples were transferred to the Biobank Service of the Laboratories of the Technologies of the Advanced Therapies of the University of Ferrara within 30 min and then immediately processed.

Samples collected in sodium citrate were centrifuged, and plasma was collected, aliquoted and frozen at −80 °C in multiple and single-use aliquots for analysis of soluble circulating factors. The biological samples of the participants were cataloged and stored anonymously using a unique alphanumeric code. After 30 min at room temperature, the blood samples were centrifuged at 1890× *g* for 10 min, and the resulting serum samples were saved at −80 °C until analysis. To measure lactate concentration, an aliquot of all serum samples was used with no further processing. This protocol for blood withdrawal and serum preparation was employed identically in both MS patients and healthy subjects. Serum levels of lactate were measured using a colorimetric L-Lactate Assay Kit according to the manufacturer’s protocol (LLactate Assay Kit Colorimetric, Abcam, ab65331). Briefly, 10 μL of serum sample plus 40 μL of lactate assay buffer was added to 96-plate wells with a reaction mix of lactate assay buffer, lactate substrate mix, and lactate enzyme mix. After incubation for 30 min, the OD at 450 nm was measured. Serum levels of pyruvate were determined using the pyruvate assay kit (Abcam, ab65342). Here, 10 μL of serum sample plus 40 μL of pyruvate assay buffer was added to 96-plate wells with a reaction mix composed of pyruvate assay buffer, pyruvate probe and enzyme mix. After incubation for 30 min, the OD at 570 nm was measured. Serum levels of glutathione were assessed by using a Glutathione Assay Kit (Abcam, ab65322), where 50 µL of deproteinized serum sample was added to 50 µL of cell lysis buffer and then added to 96-plate wells with a reaction mix composed of glutathione-S-transferase reagent and the dye monochlorobimane. After 1 h, the fluorescence emitted was registered and expressed as arbitrary fluorescence units (a.f.u.). The lactate/pyruvate (L/P) ratio was calculated by dividing the lactate values by the pyruvate values.

Resting muscular oxygen consumption (rmVO_2_): This is a valid method for assessing mitochondrial function [34]. In this study, rmVO_2_ was assessed by near-infrared spectroscopy (NIRS) using a continuous wave system (Oxymon-MK III, Artinis Medical Systems, Elst, Netherlands). With the patient lying in the supine position after 10 min of complete rest, NIRS optodes with an interoptode distance of 3.5 cm were placed along the medial gastrocnemius muscle. RmVO_2_ was performed by rapidly inflating a cuff placed around the thigh to a pressure of 60 mmHg to obtain venous occlusion for 30 s. The absolute rmVO_2_ value was calculated by the rate of increase in concentrations once the venous outflow was blocked, as previously reported [35,36]. For this study, the value of the more impaired limb (e.g., the paretic limb) was considered. Data collection and calculation were performed using the software Oxysoft 2.0.47 (Artinis Medical Systems, Netherlands).

### 2.4. Endurance Walking Capacity

The 6 min walking test was utilized for measuring the endurance walking capacity [37]. Patients were instructed to walk up and down on a 22 m walkway as far as possible in 6 min without encouragement, with the possibility to slow down and rest if necessary. The total distance walked (6 min walking distance, 6MWD) was recorded.

### 2.5. Rehabilitation Treatments

Participants in both rehabilitation groups received 12 two-hour training sessions over a six-week period. The full study protocol has been reported previously [33]. To summarize, patients in the RAGT group underwent RAGT on a Lokomat treadmill for approximately 40 min (Hocoma, Volketswil, Switzerland). For each training session, gait speed, body weight support, guidance force and the torque of the knee and hip drives were individually set, and they were progressively adjusted throughout the sessions. Patients in the conventional therapy (CT) group performed approximately 40 min of physiotherapist-assisted overground walking. Resting pauses were allowed as necessary, and the gait speed was set based on patient tolerance. For both groups, at the end of each session, the total distance walked and mean walking speed were recorded. In addition, the relative training intensity (RTI) was calculated “a posteriori.” This parameter was arbitrarily determined as the ratio between the subject’s average speed during the training sessions and the gait speed measured by the timed 25-foot walk test at baseline, or the higher speed theoretically attainable by that subject. For the RAGT group, the value obtained was corrected for body weight support (e.g., for 50% of body weight support, the value was multiplied by 0.50). Patients were categorized into a low or high training intensity groups for each treatment by dividing the patients according to a two-quantile distribution determined by the median.

### 2.6. Statistical Analysis

The data distribution was verified according to the Shapiro–Wilk test. Baseline characteristics of the two rehabilitation groups were compared with independent samples t tests, Mann–Whitney U tests or chi-square tests according to the nature and distribution of the data. Within-group differences were assessed by paired samples *t* test or Wilcoxon test, as appropriate. Between-group differences for all biomarkers were verified through a two-way analysis of variance (factors: treatment, time) or via independent sample *t* tests or Mann–Whitney U tests when comparing between-group differences at the end of treatment with respect to baseline. In addition, differences in biomarker variations according to individual training intensity were verified through independent sample *t* tests or Mann–Whitney U tests. Correlations between biomarkers were assessed with Spearman’s rho. A p value of 0.05 was considered statistically significant. Data analysis was performed with MedCalc Statistical Software version 19.2.1 (MedCalc, Ostend, Belgium).

## 3. Results

Of the 72 patients enrolled in the RAGTIME trial [32], 46 were considered in this secondary analysis. Twenty-six patients were excluded because they had interrupted rehabilitation treatment [32] or because they had missing biomarker data for at least one of the outcome collection time points. A pool of healthy subjects (*n* = 10) also underwent blood biomarker collection. The group of healthy subjects exhibited a significantly younger age with respect to MS (35 ± 10 vs. 56 ± 10 years; *p* < 0.001), but no sex or anthropometric differences were observed.

### 3.1. Mitochondrial Function Biomarkers at Baseline

When compared to healthy subjects, altered levels of serum lactate, pyruvate and GSH and the L/P ratio were measured in the MS population (Table 1). Higher lactate levels were observed in females (2.04 ± 0.49 vs. 1.62 ± 0.47 nmol/well; *p* = 0.004). No differences for any of the parameters were observed for phenotype or EDSS score.

Additionally, the rmVO_2_ of the MS population was abnormally high when compared with the reference values [36], without differences for sex, EDSS score or disease phenotype, and had values that correlated to pyruvate levels (r = −0.30; *p* = 0.045) and the L/P ratio (r = 0.27; *p* = 0.07).

### 3.2. Mitochondrial Function Biomarkers in Response to Rehabilitation

All patients included in the study safely completed the scheduled program, with significant improvements obtained for the 6MWD. The rehabilitative features, in terms of the number of rehabilitation sessions, distance walked, training speed and RTI, are reported in Table 2.

In the whole MS population, significant differences at both T1 and T2, i.e., after 6 and 12 rehabilitation sessions, respectively, with respect to baseline were observed for serum lactate, pyruvate, GSH and the L/P ratio (Table 3). Additionally, rmVO_2_ was significantly improved at T2 with respect to baseline. All mitochondrial function biomarker variations at the end of rehabilitation were correlated with their respective baseline values.

To summarize, metabolic biomarkers in all MS subjects were similarly improved after rehabilitation, with values approaching those observed in the healthy population (Supplementary Figure S1).

### 3.3. Mitochondrial Function Biomarker Response to Different Types of Rehabilitation

At baseline, the two treatment groups did not differ in terms of clinical characteristics or the parameters under study (Supplementary Table S1).

When considering within-group changes in biomarkers, significant differences over time were noted for both groups in lactate, pyruvate, and the L/P ratio, and they were noted for GSH in the RAGT group only.

In addition, rmVO_2_ showed significant variations in the RAGT group only (Table 4).

Considering the between-group comparison of changes after rehabilitation, RAGT showed significant differences with respect to CT for lactate (*p* = 0.012) and GSH levels (<0.001) and differences close to significance for the L/P ratio (*p* = 0.08) and rmVO_2_ (*p* = 0.07).

These differences were confirmed by the significant group-by-factor interaction (time and treatment) observed for lactate (F = 5.40; *p* = 0.006) and GSH (F = 7.93; *p* = 0.001), which was greater for the RAGT group.

Overall, both types of rehabilitation improved metabolic parameters in MS subjects, with an enhanced response following RAGT treatment.

### 3.4. Mitochondrial Function Biomarkers, Rehabilitative Factors and Endurance Walking Capacity

The endurance walking capacity significantly improved after both treatments without any differences (Table 4).

In the whole population, no relationships were observed between metabolic biomarkers and rehabilitative factors. However, a low or even nonsignificant correlation was observed between metabolic biomarker changes and RTI, especially for lactate (r = 0.22; *p* = 0.11) and pyruvate levels (r = −0.21; *p* = 0.15). Moreover, only RTI was significantly correlated with variations in the 6MWD at T2 (r = −0.30; *p* = 0.045). Interestingly, similar results were observed in both groups. 

**Figure 1:**
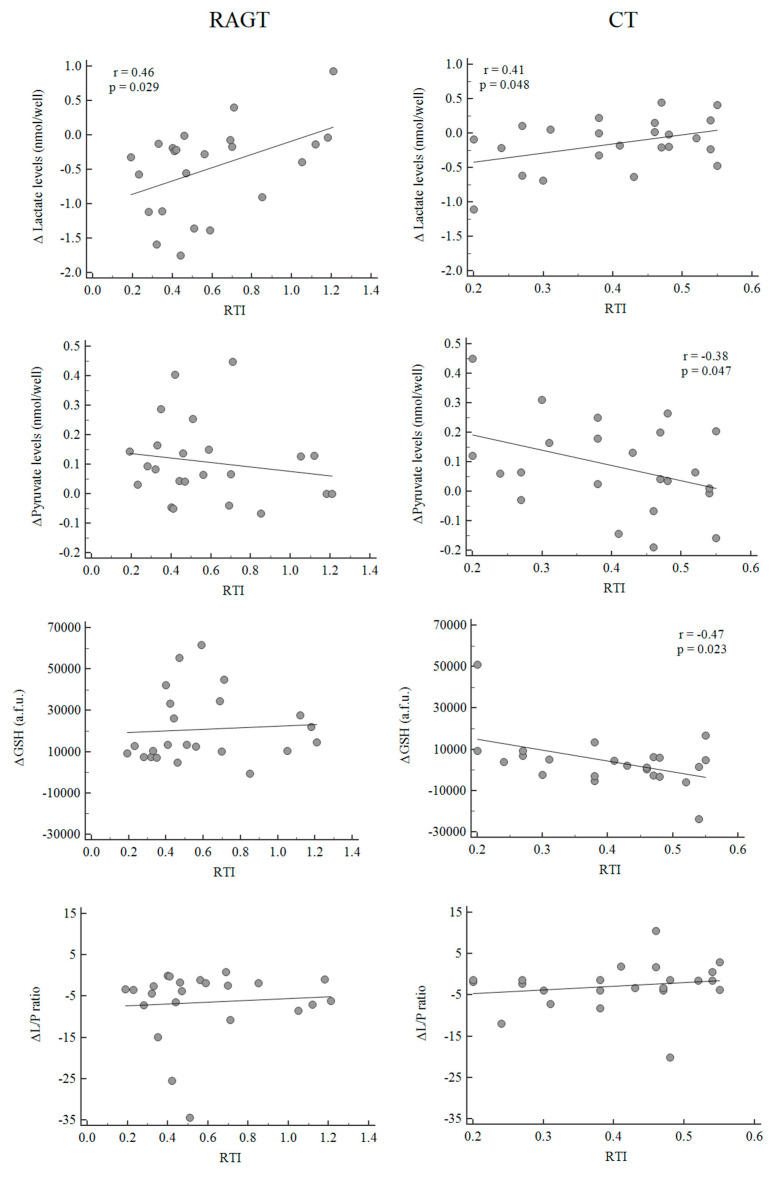
Correlation between rehabilitative factors and mitochondrial biomarkers in both groups. Abbreviations: RAGT, robot-assisted gait training; CT, conventional therapy; RTI, relative training intensity.

In the RAGT, among rehabilitative factors, a moderately significant correlation was observed only between RTI and changes in lactate levels (r = 0.46, *p* = 0.029) and pyruvate levels, although the correlation was not significant (r = −0.27; *p* = 0.12) (Figure 1). RTI was also inversely correlated with 6MWD variations (r = −0.47; *p* = 0.025).

**Figure 2:**
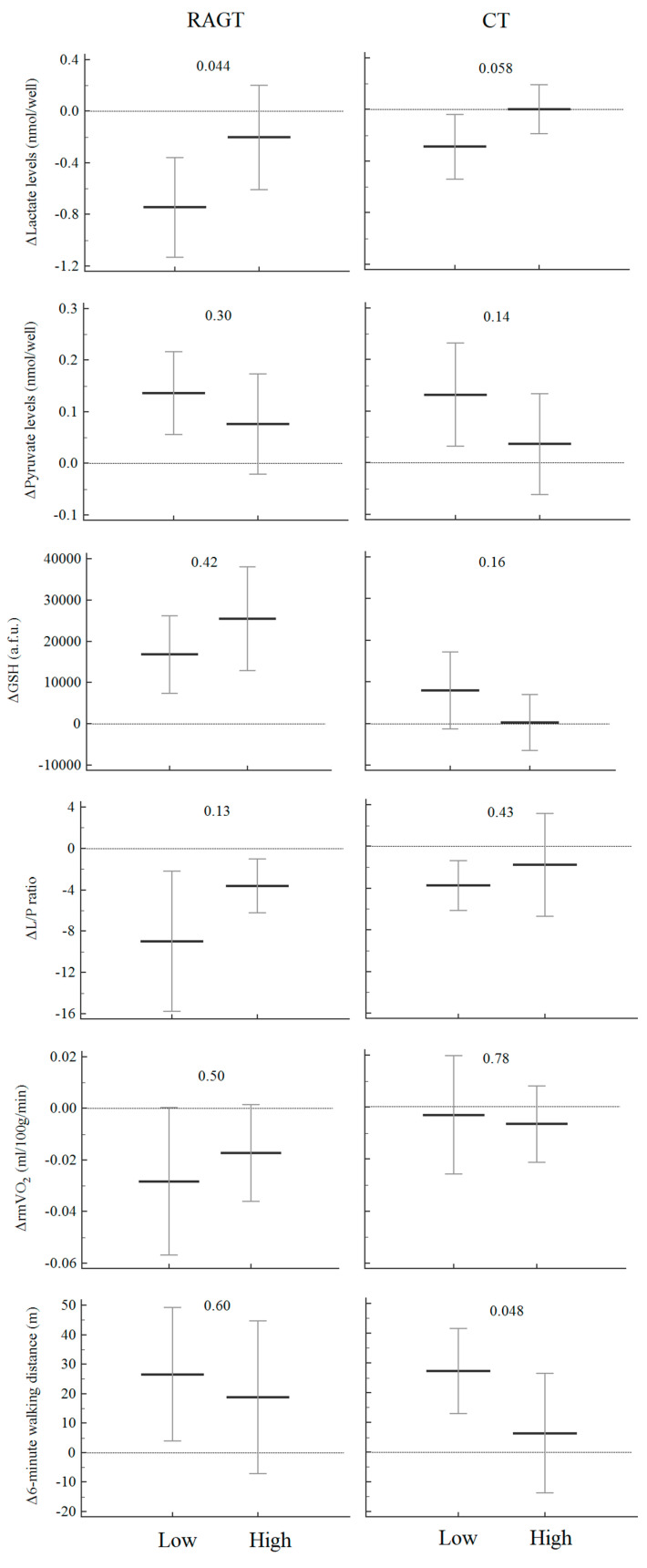
Variations of biomarkers at the end of rehabilitation compared to baseline according to two quantiles of relative training intensity in the two groups. Legend: data are expressed as median and interquartile range.

In particular, patients who exercised at low RTIs (ratio < 0.54) versus high RTIs exhibited greater improvements for lactate (*p* = 0.044) and the L/P ratio as well for 6MWD (Figure 2).

Additionally, in the CT group, significant correlations between RTI and lactate, pyruvate and GSH levels were observed (Figure 1). No correlations between endurance walking capacity and RTI were observed, despite a favorable trend (r = −0.27; *p* = 0.18).

Additionally, in this group, patients who exercised at low RTIs (ratio < 0.43) versus high RTIs exhibited greater improvements for lactate and GSH levels as well for 6MWD (*p* = 0.048) (Figure 2).

To summarize, among the rehabilitative factors, metabolic changes were exclusively correlated with RTI in both groups, with values below 50% of the spontaneous walking speed associated with metabolic and functional improvements.

## 4. Discussion

The present study showed that rehabilitative exercise in severe MS patients significantly improves energetic metabolic markers and antioxidant defense systems. In addition, the study highlights the effects of the type and intensity of rehabilitation on metabolic variables and endurance walking capacity, suggesting a novel perspective on optimal individual aerobic training.

As a first point, the study confirmed the metabolic disturbance previously reported in subjects with MS [3,9,21]. The serum lactate levels were significantly higher than those in healthy subjects, although lower than those previously reported, likely due to the diversity of methods [15]. The lactate levels at baseline were not associated with age or disease vintage but were significantly affected by sex, being higher among females. Values were only slightly correlated with EDSS scores, confirming a previous observation [15], even if the present study investigated a narrow range of EDSS scores.

In addition to lactate, MS patients showed large reductions in pyruvate amounts and altered L/P ratios. Additionally, resting muscle oxygen consumption in the gastrocnemius (a readout of skeletal muscle oxidative metabolism) was altered compared with the reference values of healthy subjects at baseline [24]. These metabolic markers strongly suggest an impairment of mitochondrial respiration, with concomitant rerouting of pyruvate into lactate reduction. Furthermore, we observed a downregulation of antioxidant defense systems as assessed by the concentration of the major intracellular antioxidant GSH [38]. As mitochondria are considered major sites of reactive oxygen species in stressed conditions, the present results support previous observations suggesting mitochondrial decline associated with MS [1,18,39].

Second, the most interesting observation is that in the whole MS population, the rehabilitative interventions were associated with a trend towards normalization for all the metabolic parameters, with a decrease in lactate levels and the L/P ratio after the end of treatment and increased pyruvate levels and GSH production over time. The changes in mitochondrial parameters were correlated with the baseline level, with a higher benefit in subjects with greater dysfunction. The same improvement was observed for resting muscle oxygen uptake, found to be abnormally high during deconditioning [35], in MS [24] and in other chronic conditions [40]. It is known that low rates of physical activity, typical of people with disabilities, trigger deconditioning with disuse-induced changes in muscle [41,42]. These changes include increased ROS production, Ca^2+^ alterations and release of proteolytic agents with fiber atrophy and reduced muscle mass [43], muscle fiber phenotype changes with marked decrease in mitochondrial enzyme activities and lower oxidative capacity [8,42,44,45], and decreases in capillary supply and blood flow in unloaded muscles [46]. The combination of unfavorable adaptive factors may also stimulate the conversion of pyruvate into lactate, even in the presence of oxygen [8] with greater lactate and H+ accumulation during exercise [42].

It is also well known that the effects of deconditioning can be improved or reversed by exercise. In elderly and young people, 14 days of exercise training reversed a decrease in key regulators of mitochondrial biogenesis, which were downregulated after bed rest, with a shift from type IIx to IIa fibers after training [47,48]. A relatively higher aerobic fiber type proportion was observed after twelve weeks of training programs at different intensities in MS patients [49]. Compared to this study, the rapid change in metabolic parameters that we measured after only two weeks of training may be due to the degree of physical activity restriction and the consequent degree of deconditioning at baseline related to the clinical status (EDSS average 6.3 vs. 2.7) and/or the effects of the different modes of exercise (walking instead of cycling) [49]. As a further effect of activation following rehabilitation, the increased levels of pyruvate, a potent scavenger of ROS [5,12,13], as well as the positive effect on antioxidant defense may represent a hallmark of reduced inflammatory status, which is significant in neuropathologies [5].

Interestingly, in our study, a further novel observation was the different patterns of metabolic response observed for the two treatments. Patients following both treatments showed significantly and progressively improved mitochondrial metabolism and simultaneously reduced oxidative stress (GSH production) over time, with superior effects for RAGT. Among the rehabilitative factors, the changes in lactate and pyruvate in particular were only related to a novel parameter determined by a posteriori analysis: relative training intensity. Indeed, walking speed and other therapy setting factors may influence the ability of RAGT to induce cardiometabolic and neuromuscular changes [50]. The training load during rehabilitation in general is not always individually prescribed on the basis of FITT (frequency, intensity, time and type) principles [51] but is routinely administered according to the therapist’s experience and/or the patient’s feelings [52] or perception of fatigue [53], a subjective parameter that is difficult to objectivize [50].

In the RAGTIME study, we preliminarily observed that patients were trained at a different (lower or higher) walking speed in relation to their degree of mobility at baseline, and we reported that the “responders” to each treatment walked at a lower speed [30]. In this study, to objectively compare the relative intensity of training for each patient, we arbitrarily calculated the ratio between the average training speed performed and the higher exercise intensity theoretically attainable by each patient or the timed 25-foot walk test speed recorded at baseline. This analysis showed that subjects who walked at a slower individual speed, i.e., below 50% of the spontaneous walking speed, improved their metabolic pattern, independent of sex, age, phenotype and allocation group. However, the best response, in terms of reduction of mitochondrial metabolism and oxidative damage, which was clearly observed in the RAGT group, could be linked to facilitation of the conversion of muscle fibers towards relatively aerobic components or to a lower energy and mechanical muscle stress related to RAGT rather than overground walking.

If RTI was involved in metabolic restoration, this parameter was also associated with changes in endurance walking capacity. In both groups, the 6MWD, which showed similar improvement after training, was selectively improved following low RTI training. The personalized intensity of exercise administered to the patients might contribute to addressing the problem of the complex and person-specific response to rehabilitation [54]. Different peripheral adaptations might be the result of different training intensities considering that the higher the intensity, the more type 2 fibers are recruited [21]. Again, according to the training speed, stimulation of conversion of fibers might be different in terms of the proportion of I and II fibers, as previously reported in MS subjects training at different intensities [49]. Improved 6MWDs were observed in MS subjects with the same disability level following low intensity interval walking associated with mild blood flow restriction in the lower limb muscles [55].

The study has several limitations. The reference population was younger than the patient population. The reduced sample of patients might have caused selection bias with respect to the RAGTIME trial. RTI does not allow us to compare the absolute intensity and the cardiovascular load of the two treatments because physiological responses related to each intervention (oxygen consumption, heart rate and perceived exertion) were not measured. The cut-off value of relative intensity was mathematically calculated “a posteriori”. Finally, the data focus on the response to a chronic training program without addressing the importance of the persistence of the effect.

## 5. Conclusions

In conclusion, rehabilitation improved altered mitochondrial energetics and reduced oxidative stress in severely disabled MS patients. In the study, the effect was rapid, particularly evident in the presence of higher metabolic dysfunction and observable in both groups, and even more pronounced following RAGT training. In both groups, a training intensity lower than that normally tolerated and administered was accompanied by higher metabolic and functional improvements.

The study, which supports the importance of muscle oxidative capacity in the walking performance of MS subjects [56], suggests that personalized training could improve the effectiveness of rehabilitation and reduce heterogeneous mobility outcomes across individuals. 

In addition, a training based on the FITT principles, individualized using a parameter easily determinable at baseline, might improve the effectiveness of RAGT and the metabolic status. When confirmed in further long-term studies, this knowledge could be applicable in future rehabilitative interventions.

## Figures and Tables

**Table 1:** Differences in biomarkers at baseline between MS patients and a sample of healthy subjects.

	MS (*n* = 46)	Healthy (*n* = 10)	*p*
Lactate, nmol/well	1.89 ± 0.52	1.24 ± 0.76	0.001
Pyruvate, nmol/well	0.26 ± 0.12	0.65 ± 0.18	<0.001
GSH, a.f.u.	40,896 ± 21,968	84,420 ± 25,181	<0.001
Lactate/Pyruvate ratio	9.55 ± 7.12	1.75 ± 0.73	0.001

Abbreviations: MS, multiple sclerosis; GSH, glutathione; a.f.u. arbitrary fluorescence units.

**Table 2:** Rehabilitative factors for the whole population and the two groups.

	Whole Population (*n* = 46)	RAGT (*n* = 23)	CT (*n* = 23)	*p*
Sessions, n	11.7 ± 0.7	11.8 ± 0.7	11.7 ± 0.7	0.53
Average walking speed, kmh^−1^	1.3 ± 0.4	1.8 ± 0.1	0.8 ± 0.6	<0.001
Total distance walked, m	8162 ± 3664	10217 ± 1109	6108 ± 4171	<0.001
Body weight support, %	44 ± 8	44 ± 8	n.a.	n.a.
Relative Training Intensity *, a.u.	0.54 ± 0.33	0.68 ± 0.41	0.40 ± 0.11	0.003

Abbreviations. a.u.: arbitrary units. Legend. * relative training intensity calculation: ((average training speed/baseline gait speed) * ((100 − body weight support)/100)).

**Table 3:** Values of the outcome parameters at the different time points of collection.

	T0	T1	T2
Lactate, nmol/well	1.89 (1.73–2.04)	1.75 * (1.61–1.89)	1.52 * † (1.37–1.68)
Pyruvate, nmol/well	0.26 (0.22–0.30)	0.34 * (0.30–0.37)	0.35 * (0.32–0.39)
GSH, a.f.u.	40,896 (34,373–47,420)	43,713 * (36,747–50,678)	52,859 * † (46,002–59,716)
Lactate/pyruvate ratio	9.55 (7.44–11.67)	5.89 * (5.09–6.86)	4.93 * † (4.11–5.75)
rmVO_2_, mlO_2_/min/100 g	0.06 (0.05–0.07)	0.06 (0.04–0.07)	0.05 * (0.04–0.06)
6MWD, m	150 (121–180)	164 * (134–194)	170 * (137–204)

Abbreviations: GSH, glutathione; a.f.u. arbitrary fluorescence units; rmVO_2_, resting muscle oxygen consumption; 6MWD, 6 min walking distance. Legend: Data are reported as mean (95% confidence interval). Within-group comparison: * *p* < 0.05 with respect to T0; † *p* < 0.05 with respect to T1.

**Table 4:** Values of the biomarkers at the different time points of collection in both groups.

	RAGT (*n* = 23)			CT (*n* = 23)		
	T0	T1	T2	T0	T1	T2
Lactate, nmol/well	2.00 (1.76–2.23)	1.71 * (1.52–1.91)	1.42 * † (1.19–1.66)	1.77 (1.57–1.98)	1.79 (1.58–2.00)	1.63 * † (1.41–1.85)
Pyruvate, nmol/well	0.26 (0.21–0.32)	0.37 * (0.31–0.42)	0.38 * (0.33–0.42)	0.26 (0.21–0.31)	0.31 * (0.26–0.36)	0.33 * (0.27–0.38)
GSH, a.f.u.	39,566 (30,487–48,645)	45,214 (34,746–55,681)	59,286 * † (50,688–67,884)	42,227 (32,154–52,300)	42,212 (32,212–52,212)	46,432 (35,762–57,102)
Lactate/pyruvate ratio	10.59 (6.87–14.30)	5.30 * (4.14–6.46)	4.16 * † (3.33–5.00)	8.52 (6.25–10.78)	6.48 * (5.35–7.59)	5.70 * (4.29–7.11)
rmVO2, mlO_2_/min/100 g	0.07 (0.06–0.09)	0.06 (0.04–0.08)	0.05 * (0.04–0.06)	0.06 (0.04–0.07)	0.05 (0.03–0.07)	0.05 (0.03–0.07)
6MWD, m	151 (114–187)	168 * (129–207)	173 * (127–218)	150 (100–200)	160 * (111–210)	167 * (114–220)

Abbreviations: RAGT, robot-assisted gait training; CT, conventional therapy; GSH, glutathione; a.f.u. arbitrary fluorescence units; rmVO_2_, resting muscle oxygen consumption; 6MWD, 6 min walking distance. Legend: Data are reported as mean (95% confidence interval). Within-group comparison: * *p* < 0.05 with respect to T0; † *p* < 0.05 with respect to T1.

## Supplementary Materials

The following are available online at https://www.mdpi.com/2075-4418/10/10/834/s1. Figure S1. Values of mitochondrial biomarkers and endurance walking capacity at the three time points of collection for the entire population. Table S1. Clinical characteristics of patients randomized in the two treatment groups.
