# Supplementary material for: Rehabilitation Improves Mitochondrial Energetics in Progressive Multiple Sclerosis: The Significant Role of Robot-Assisted Gait Training and of the Personalized Intensity
Source: Diagnostics (Basel). 2020 Oct 17;10(10):834. doi: 10.3390/diagnostics10100834 (PMC7602995; doi:10.3390/diagnostics10100834)
Supplement: Supplementary file 1 [file diagnostics-10-00834-s001.pdf]

diagnostics

MDPI

Supplementary

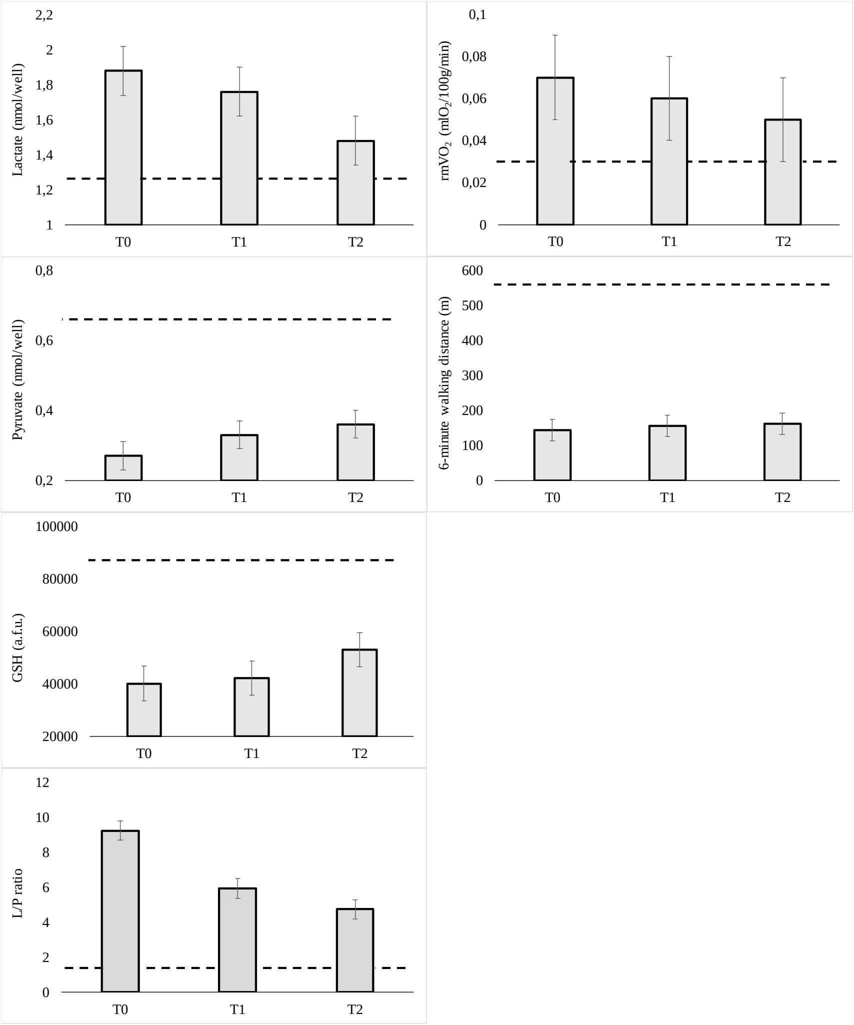

Figure S1. Values of mitochondrial biomarkers and endurance walking capacity at the three time points of collection for the entire population. Black dashed line represent healthy population mean value.

Table S1. Clinical characteristics of patients randomized in the two treatment groups.

	RAGT (n=23)	CT (n=23)	Between-group p
Age, years	57 ±9	55 ±11	0.55
Male sex, n(%)	6 (26)	11 (48)	0.23
MS duration, years	14 ±10	19 ±11	0.11
EDSS	6.3 ±0.3	6.4 ±0.3	0.44
Primary progressive, n(%)	12 (52)	9 (39)	0.38
Secondary progressive, n(%)	11 (48)	14 (61)	0.38
Disease modifying therapy, n(%)	4 (17)	5 (22)	0.81
Symptomatic therapy, n(%)	7 (30)	9 (40)	0.42

Abbreviations: RAGT, robot-assisted gait training; CT, conventional therapy; MS, multiple sclerosis; EDSS, expanded disability status scale.
